# Epidemiology and risk factors for *Staphylococcus aureus *colonization in children in the post-PCV7 era

**DOI:** 10.1186/1471-2334-9-110

**Published:** 2009-07-11

**Authors:** Grace M Lee, Susan S Huang, Sheryl L Rifas-Shiman, Virginia L Hinrichsen, Stephen I Pelton, Ken Kleinman, William P Hanage, Marc Lipsitch, Alexander J McAdam, Jonathan A Finkelstein

**Affiliations:** 1 Department of Population Medicine, Harvard Pilgrim Health Care Institute and Harvard Medical School, Boston, MA, USA; 2 Division of Infectious Diseases, Children's Hospital Boston, Boston, MA, USA; 3 Department of Laboratory Medicine, Children's Hospital Boston, Boston, MA, USA; 4 Division of Infectious Diseases, University of California Irvine School of Medicine, Irvine, CA, USA; 5 Section of Pediatric Infectious Diseases, Boston Medical Center, Boston, MA, USA; 6 Department of Infectious Disease Epidemiology, Imperial College, London, UK; 7 Department of Epidemiology, Harvard School of Public Health, Boston, MA, USA; 8 Division of General Pediatrics, Children's Hospital Boston, Boston, MA, USA

## Abstract

**Background:**

The incidence of community-associated methicillin-resistant *Staphylococcus aureus *(MRSA) has risen dramatically in the U.S., particularly among children. Although *Streptococcus pneumoniae *colonization has been inversely associated with *S. aureus *colonization in unvaccinated children, this and other risk factors for *S. aureus *carriage have not been assessed following widespread use of the heptavalent pneumococcal conjugate vaccine (PCV7). Our objectives were to (1) determine the prevalence of *S. aureus *and MRSA colonization in young children in the context of widespread use of PCV7; and (2) examine risk factors for *S. aureus *colonization in the post-PCV7 era, including the absence of vaccine-type *S. pneumoniae *colonization.

**Methods:**

Swabs of the anterior nares (*S. aureus*) were obtained from children enrolled in an ongoing study of nasopharyngeal pneumococcal colonization of healthy children in 8 Massachusetts communities. Children 3 months to <7 years of age seen for well child or sick visits in primary care offices from 11/03–4/04 and 10/06–4/07 were enrolled. *S. aureus *was identified and antibiotic susceptibility testing was performed. Epidemiologic risk factors for *S. aureus *colonization were collected from parent surveys and chart reviews, along with data on pneumococcal colonization. Multivariate mixed model analyses were performed to identify factors associated with *S. aureus *colonization.

**Results:**

Among 1,968 children, the mean age (SD) was 2.7 (1.8) years, 32% received an antibiotic in the past 2 months, 2% were colonized with PCV7 strains and 24% were colonized with non-PCV7 strains. The prevalence of *S. aureus *colonization remained stable between 2003–04 and 2006–07 (14.6% vs. 14.1%), while MRSA colonization remained low (0.2% vs. 0.9%, p = 0.09). Although absence of pneumococcal colonization was not significantly associated with *S. aureus *colonization, age (6–11 mo vs. ≥5 yrs, OR 0.39 [95% CI 0.24–0.64]; 1–1.99 yrs vs. ≥5 yrs, OR 0.35 [0.23–0.54]; 2–2.99 yrs vs. ≥5 yrs, OR 0.45 [0.28–0.73]; 3–3.99 yrs vs. ≥5 yrs, OR 0.53 [0.33–0.86]) and recent antibiotic use were significant predictors in multivariate models.

**Conclusion:**

In Massachusetts, *S. aureus *and MRSA colonization remained stable from 2003–04 to 2006–07 among children <7 years despite widespread use of pneumococcal conjugate vaccine. *S. aureus *nasal colonization varies by age and is inversely correlated with recent antibiotic use.

## Background

Infections due to *Staphylococcus aureus *are a growing clinical and public health problem in the US. [1-3]*S. aureus *can cause skin and soft tissue infections, pneumonia, bloodstream infections, and bone and joint infections in both children and adults. [4-12] Treatment of these infections has become more difficult in recent years due to the emergence and rapid spread of drug-resistant strains such as methicillin-resistant *S. aureus *(MRSA). Most troubling is the dramatic increase in community-associated MRSA (CA-MRSA) infections now being reported in healthy children and adults, but little is known about how patterns of colonization with *S. aureus *in the community have changed. [1,13-15]

Colonization of the anterior nares by *S. aureus *has been shown to be associated with subsequent infection in an individual. [16,17] Furthermore, host and/or bacterial factors may increase this risk, such that a high fraction of colonized persons become infected. [18,19] For example, a natural history study in a military population demonstrated that soft-tissue infections occurred more frequently after colonization with CA-MRSA in comparison to methicillin-susceptible *S. aureus *(38% vs. 3%). [18] Given the risk of infection associated with colonization, particularly with CA-MRSA strains, a greater understanding of the epidemiology of *S. aureus *colonization is needed in order to determine optimal control and prevention strategies.

Colonization with vaccine-type *S. pneumoniae *in the nasopharynx has been shown to be inversely associated with *S*. *aureus *nasal carriage in unvaccinated populations. [20,21] This association is of particular concern given universal immunization with pneumococcal conjugate vaccine in the U.S. and the finding of replacement of vaccine serotypes with non-vaccine serotypes in the nasopharynx. [22] If vaccine serotypes do indeed interfere with colonization by *S*. *aureus*, use of PCV7 and the resulting removal of vaccine-type pneumococci from carriage may result in increased mucosal colonization with *S*. *aureus*.

Further elucidation of the epidemiology of *S*. *aureus *colonization in young children, particularly in vaccinated populations, and identification of risk factors for colonization is needed for the development of strategies for prevention in the community. In this article we take advantage of a unique opportunity to examine the prevalence and risk factors for *S*. *aureus *colonization in the context of an ongoing study of nasopharyngeal pneumococcal colonization among healthy children in Massachusetts. Our objectives were to: (1) determine the prevalence of *S*. *aureus *and MRSA colonization in healthy children in the post-PCV7 era and (2) examine risk factors for *S*. *aureus *colonization, including the absence of vaccine-type *S. pneumoniae *colonization.

## Methods

### Study Design and Population

We conducted a prospective observational study of *S. aureus *colonization among healthy children in Massachusetts. [23] This study was nested within an existing study to assess serial changes in *S. pneumoniae *serotypes and antibiotic resistance in the post-PCV7 era. [24] Children 6 years and younger who were seen for either well-child care visits or sick care visits in participating primary care practices were eligible for this study. Practices were selected from 16 communities in Massachusetts in 2003–04 and from 8 of these original 16 communities in 2006–07. Each participating child was sampled, at most, once during each swabbing season during 11/03–4/04 and 10/06–4/07. Written informed consent was obtained from parents. This study was approved by the Harvard Pilgrim Health Care Institutional Review Board.

Parents of enrolled children completed a written survey at the time of the visit that included information on age, gender, race/ethnicity, history of being breastfed, childcare attendance (>4 hours/week), smokers in the home, family members who work in a hospital or long-term care facility, recent antibiotic use, and history of chronic illness in children. Dates of immunization with PCV7 and influenza vaccines, diagnoses on day of visit, type of antibiotics received in the past 2 months, and patient zip code were obtained from medical record review. Because data on individual household income and educational level were not uniformly available, we used median census tract household income and educational level from the 2000 U.S. census based on geocoding address data.

### Specimen Collection and Processing

Anterior nares swabs were collected by introducing a sterile calcium alginate-tipped swab on an aluminum shaft and gently rotating the swab. Swabs were transported by overnight courier and processed within 24 hours. Specimens were swabbed onto mannitol salt agar (MSA) plates where they were incubated at 37°C for 48 hours. Plates were examined for the presence of Mannitol-fermenting colonies daily and these were subcultured to trypticase soy agar and 5% sheep blood agar plates (BAPs) and incubated at 37°C overnight. Overnight cultures on BAPs were tested by catalase, Gram stain and Staphaurex (Remel, Lenexa, Kansas), a rapid latex-agglutination kit for identifying *S. aureus*. Catalase positive, Gram positive cocci which were negative by Staphaurex were tested for coagulase using rabbit plasma with EDTA. Catalase positive Gram-positive cocci in clusters which were positive in either the Staphaurex or coagulase tests were identified as *S. aureus *and underwent sensitivity testing by either disk diffusion or E-test for the following antibiotics: penicillin, oxacillin, erythromycin, clindamycin, trimethoprim/sulfamethoxazole, tetracycline, vancomycin, linezolid and mupirocin. For erythromycin-resistant, clindamycin-susceptible isolates, a D-test was also performed to determine the presence of erythromycin-inducible clindamycin resistance. *S. aureus *isolates were considered mupirocin resistant if the MIC was >4 μg/ml. [25] Isolation of *S. pneumoniae *from NP swabs, serotyping, and antimicrobial susceptibility testing were performed as described previously. [23]

### Data Analysis

We calculated the prevalence of *S. aureus *and MRSA colonization as a percentage with 95% confidence intervals. When assessing trends in *S. aureus *colonization over time, we compared the prevalence of colonization between 2003–04 and 2007–08 for the 8 communities participating in both sampling periods. To examine the relationship between *S. pneumoniae *and *S. aureus *co-colonization in individual children, we utilized data from all 16 communities. Categorical variables were compared using a chi-square test.

To assess risk factors for *S. aureus *colonization, multivariable logistic regression models were used. Clustering within communities was accounted for using generalized linear mixed models. [26,27] Models of *S. aureus *colonization included the following variables: (1) year of swab, individual and community-level demographic characteristics, (2) *S. pneumoniae *colonization, (3) recent antibiotic use (within 2 months), (4) breastfeeding, (5) use of childcare outside the home, (6) diagnosis of illness on day of swab, (7) number of doses of PCV7 received, and (8) recent influenza vaccination. For the risk factor analyses, data from all available communities in each sampling period were used in our primary analyses. We also performed secondary analyses limited to the 8 communities surveyed in both periods. Because our results did not differ when analyses were limited to the 8 communities, results from our primary analyses are presented here. All analyses were performed using SAS version 9.1.3 (SAS Institute, Cary, NC).

## Results

### Study population

One thousand nine hundred sixty-eight children were enrolled in the study during 2003–04 and 2006–07, combined. The mean (SD) age of children was 2.7 (1.8) years with range of 0.3 to <7.0 years (Table 1). Children were mostly white non-Hispanic (83.6%) and about half (53.2%) lived in communities where the median annual household income, based on U.S. census data for 2000, was less than $55,000. Children were enrolled in at least 4 hours of childcare per week (50.7%), had smokers in the home (19.9%), had a family member work in a hospital or long-term care facility (17.4%), received an antibiotic in the past 2 months (32.2%), received 3 or more doses of PCV7 vaccine (70.6%), and received influenza vaccine in the same season prior to their visit (24.0%).

**Table 1:** Characteristics of the study population.

	Total N = 1,968)	16 communities swabbed in 2003–04 (N = 994)		8 communities swabbed in 2006–07 (N = 974)
		
		8 communities swabbed in 2003–04 only (N = 406)	8 communities swabbed in both seasons (N = 588)	8 communities swabbed in both seasons (N = 974)
Age group (N = 1,968)				
3–<6 months	131 (6.7%)	25 (6.2%)	30 (5.1%)	76 (7.8%)
6–<12 months	299 (15.2%)	53 (13.1%)	67 (11.4%)	179 (18.4%)
1–<2 years	477 (24.2%)	100 (24.6%)	129 (21.9%)	248 (25.5%)
2–<3 years	274 (13.9%)	61 (15.0%)	82 (14.0%)	131 (13.5%)
3–<4 years	237 (12.0%)	57 (14.0%)	84 (14.3%)	96 (9.9%)
4–<5 years	231 (11.7%)	50 (12.3%)	79 (13.4%)	102 (10.5%)
5–<7 years	319 (16.2%)	60 (14.8%)	117 (19.9%)	142 (14.6%)

Female (N = 1,968)	981 (49.9%)	216 (53.2%)	308 (52.4%)	457 (46.9%)

Race/ethnicity (N = 1,872)				
White non-Hispanic	1,565 (83.6%)	341 (83.9%)	463 (84.0%)20 (3.6%)	761 (82.4%)
Black non-Hispanic	86 (4.6%)	15 (3.8%)	20 (3.6%)	51 (5.5%)
Hispanic	122 (6.5%)	23 (5.8%)	28 (5.1%)	71 (7.7%)
Other	99 (5.3%)	18 (4.5%)	40 (7.3%)	41 (4.4%)

Median household income in community (N = 1,869)*				
<40,000	373 (20.0%)	125 (31.8%)	104 (18.4%)	144 (15.8%)
40,000–<55,000	620 (33.2%)	122 (31.0%)	191 (33.9%)	307 (33.7%)
55,000–<70,000	447 (23.9%)	85 (21.6%)	135 (23.9%)	227 (24.9%)
≥70,000	429 (23.0%)	61 (15.5%)	134 (23.8%)	234 (26.7%)

% community members with <high school educational level (N = 1,869)*				
<5%	297 (15.9%)	57 (14.5%)	79 (14.0%)	161 (17.7%)
5–<10%	387 (20.7%)	95 (24.2%)	111 (19.7%)	181 (19.9%)
10–<15%	485 (26.0%)	66 (16.8%)	164 (29.1%)	255 (28.0%)
≥15%	700 (37.5%)	175 (44.5%)	210 (37.2%)	315 (34.5%)

Ever breastfed (N = 1,900)	1,291 (68.0%)	237 (59.4%)	400 (69.8%)	654 (70.5%)

Childcare or school for ≥4 hours (N = 1,857)	941 (50.7%)	184 (46.8%)	296 (53.9%)	461 (50.4%)

Smokers in home (N = 1,784)	355 (19.9%)	97 (25.5%)	87 (16.1%)	171 (19.9%)

Family member works in hospital or LTCF (N = 1,888)	328 (17.4%)	66 (16.7%)	98 (17.5%)	164 (17.6%)

Antibiotics in past 2 months (N = 1,945)				
Amoxicillin-clavulanate/cephalosporins/mupirocin/TMP-SMX	212 (10.9%)	42 (10.8%)	61 (10.5%)	109 (11.2%)
Clindamycin/macrolides	82 (4.2%)	22 (5.7%)	24 (4.1%)	36 (3.7%)
Amoxicillin/penicillin	332 (17.1%)	74 (19.0%)	86 (14.8%)	172 (17.7%)
None	1,319 (67.8%)	251 (64.5%)	411 (70.6%)	657 (67.5%)

Antibiotics in past 6 months for skin infection (N = 1,950)	29 (1.5%)	0 (0%)	1 (0.2%)	28 (2.9%)

History of chronic illness				
Asthma/RAD (N = 1,950)	199 (10.2%)	27 (6.9%)	80 (13.7%)	92 (9.5%)
Skin condition† (N = 974)	47 (4.8%)	-	-	47 (4.8%)

Diagnosis on day of visit (N = 1,950)				
Asthma/RAD	73 (3.7%)	18 (4.6%)	19 (3.3%)	36 (3.7%)
Rash	65 (3.3%)	9 (2.3%)	12 (2.1%)	44 (4.5%)
Otitis media	258 (13.2%)	30 (7.7%)	61 (10.5%)	167 (17.2%)
Respiratory tract infection	461 (23.6%)	76 (19.4%)	95 (16.3%)	290 (29.8%)
Skin/soft tissue infection	14 (0.7%)	0 (0%)	1 (0.2%)	13 (1.3%)
Allergic rhinitis/seasonal allergies	13 (0.7%)	3 (0.8%)	7 (1.2%)	3 (0.3%)

Colonization with *Streptococcus pneumoniae *(N = 1,964)	524 (26.7%)	91 (22.4%)	141 (24.0%)	292 (30.1%)

Pneumococcal vaccination** (N = 1,950)				
0 doses	209 (10.7%)	76 (19.4%)	113 (19.4%)	20 (2.1%)
1 dose	170 (8.7%)	40 (2.1%)	61 (3.1%)	69 (7.1%)
2 doses	194 (10.0%)	38 (9.7%)	82 (14.0%)	74 (7.6%)
3 doses	1377 (70.6%)	238 (60.7%)	328 (56.2%)	811 (83.3%)

Influenza vaccination prior to visit††(N = 1,950)	467 (24.0%)	65 (16.6%)	45 (7.7%)	357 (36.7%)

*Household income and educational level based on geocoded information from 2000 U.S. census.

† Assessed in 2007–08 season only

** Pneumococcal dose included if given at least 30 days prior to swab

†† Flu vaccination in that season at least 2 weeks prior to visit

### Staphylococcus aureus colonization

14.6% (95% CI, 11.9%–17.7%) of children were colonized with *S. aureus *in 2003–04 vs. 14.1% (95% CI, 11.9%–16.4%) in 2006–07 (p = 0.68) in the 8 communities swabbed during both periods, whereas 16.3% of children were colonized in the 8 communities swabbed during 2003–04 only. MRSA colonization in healthy children remained low at 0.2% (95% CI, 0.0%–0.9%) in 2003–04 and 0.9% (95% CI, 0.4%–1.7%) in 2006–07 (p = 0.09) for 8 communities, compared to 0.7% (95% CI 0.2%–2.1%) for the other 8 communities swabbed only during 2003–04. Antibiotic susceptibilities for *S. aureus *isolates for the 8 communities swabbed in 2003–04 and 2006–07 were as follows: penicillin (11% vs. 9%), erythromycin (80% vs. 72%), clindamycin (98% vs. 80%, p < 0.001), trimethoprim/sulfamethoxazole (100% vs. 100%), tetracycline (98% vs. 98%), mupirocin (99% vs. 99%), vancomycin (100% vs. 100%), and linezolid (100% vs. 100%) in the 8 communities swabbed during both periods. Of these, 10 MRSA isolates were identified with 0 susceptible to erythromycin, 4 susceptible to clindamycin, 9 susceptible to tetracycline, and all susceptible to trimethoprim/sulfamethoxazole, mupirocin, vancomycin and linezolid.

### Staphylococcus aureus and Streptococcus pneumoniae colonization

Between 2003–04 and 2006–07, colonization with PCV7 strains declined (3.4% vs. 1.0%, p < 0.001) while colonization with non-PCV7 strains increased (19.4% vs. 29.1%, p < 0.001) among children enrolled in the study. No significant difference in colonization with *S. aureus *was noted among those children who were and were not colonized with *S. pneumoniae*. (12.4% vs. 15.5%; p = 0.10). To explore whether non-PCV7 carriage differentially affected *S. aureus *carriage, we further examined the proportion of people colonized with PCV7 strains vs. non-PCV7 strains compared to those who were not colonized with pneumococcus (Table 2). In children colonized with PCV7 or non-PCV7 strains, 11.4% and 12.7% were also colonized with *S. aureus*, respectively, compared to 15.5% for children who were not colonized with pneumococci (p = 0.29).

**Table 2:** *Streptococcus pneumoniae *and *Staphylococcus aureus *colonization.

	*S. aureus *colonization	
	**No**	**Yes**

**PCV7 strains**	39 (88.6%)	5 (11.4%)

**Non-PCV7 strains**	414 (87.3%)	60 (12.7%)

**No *S. pneumoniae *colonization**	1217 (84.5%)	223 (15.5%)

**Total**	1670 (85.3%)	288 (14.7%)

Unadjusted p-value = 0.29

### Risk factors for colonization

We examined risk factors for *S. aureus *colonization in children <7 years (Table 3). Bivariate analyses revealed that age had a U-shaped relationship with *S. aureus *colonization, with increased colonization among children <6 months and 5–<7 years. *S. aureus *colonization was lowest among children 1–<2 years of age. Use of antibiotics in the past 2 months was also associated with lower risk of *S. aureus *colonization. When the type of antibiotics used in the past 2 months was considered, we found that the inverse relationship with *S. aureus *colonization remained regardless of whether the antibiotic was very likely (amoxicillin-clavulanate, cephalosporins, mupirocin, trimethoprim-sulfamethoxazole), somewhat likely (clindamycin, macrolides), or unlikely (amoxicillin, penicillin) to have clinical activity against *S. aureus*.

**Table 3:** Proportion colonized with *Staphylococcus aureus *and bivariate predictors of colonization adjusted for clustering by community

	% colonized	Bivariate OR (95% CI)	P-value
Year of Swab (N = 1,968)			0.43
2003–04	15.3%	1.0	
2006–07	14.1%	0.90 (0.70–1.17)	

Age group (N = 1,968)			<0.001
<6 months	16.8%	0.59 (0.35–1.00)	
6–<12 months	10.7%	0.36 (0.23–0.55)	
1–<2 years	9.4%	0.31 (0.21–0.46)	
2–<3 years	12.0%	0.40 (0.26–0.63)	
3–<4 years	13.9%	0.47 (0.30–0.74)	
4–<5 years	18.6%	0.68 (0.45–1.03)	
≥5 years	25.4%	1.0	

Female (N = 1,968)	13.7% vs. 15.7%	0.85 (0.66–1.09)	0.20

Race/ethnicity (N = 1,872)			0.85
White non-Hispanic	14.3%	1.0	
Black non-Hispanic	16.3%	1.15 (0.64–2.08)	
Hispanic	15.6%	1.08 (0.65–1.80)	
Other	12.1%	0.81 (0.44–1.51)	

Median household income in community (N = 1,869)			0.76
<$40,000	12.9%	0.83 (0.54–1.27)	
$40,000–$54,999	15.3%	1.01 (0.70–1.45)	
$55,000–$69,999	14.1%	0.92 (0.62–1.35)	
≥$70,000	15.4%	1.0	

% community members with <high school educational level (N = 1,869)			0.74
<5%	16.2%	1.10 (0.74–1.64)	
5–<10%	13.4%	0.88 (0.61–1.28)	
10–<15%	13.8%	0.92 (0.66–1.29)	
≥15%	15.0%	1.0	

Ever breastfed (N = 1,900)	14.5% vs. 14.6%	0.99 (0.75–1.30)	0.94

Childcare or school for ≥4 hrs (N = 1,857)	15.7% vs. 13.1%	1.24 (0.95–1.60)	0.11

Smokers in home (N = 1,784)	15.5% vs. 13.8%	1.16 (0.83–1.60)	0.39

Family member works in hospital or long-term care facility (N = 1,888)	11.9% vs. 15.0%	0.76 (0.53–1.09)	0.14

Antibiotics in past 2 months (N = 1,945)			<0.001
Amoxicillin-clavulanate/cephalosporins/mupirocin/TMP-SMX	8.0%	0.42 (0.25–0.71)	
Clindamycin/macrolides	9.8%	0.51 (0.24–1.08)	
Amoxicillin/penicillin	10.5%	0.57 (0.39–0.83)	
No antibiotics	17.1%	1.0	

Antibiotics in past 6 months for a skin infection (N = 1,950)	10.3% vs. 14.7%	0.67 (0.20–2.22)	0.51

History of chronic illness (N = 1,950)			
Asthma/RAD (N = 1,950)	17.6% vs. 14.3%	1.29 (0.88–1.91)	0.20
Skin condition (N = 974)	17.0% vs. 13.9%	1.27 (0.58–2.78)	0.55

Diagnosis on day of visit (N = 1,950)			
Asthma/RAD	20.6% vs. 14.3%	1.57 (0.88–2.81)	0.13
Rash	21.5% vs. 14.4%	1.66 (0.91–3.03)	0.10
Otitis media	12.0% vs. 15.0%	0.77 (0.52–1.14)	0.19
Respiratory tract infection	16.5% vs. 14.0%	1.20 (0.90–1.59)	0.22
Skin/soft tissue infection	7.1% vs. 14.7%	0.43 (0.06–3.29)	0.42
Allergic rhinitis/seasonal allergies	7.7% vs. 14.7%	0.48 (0.06–3.70)	0.48

Colonization with *Streptococcus pneumoniae *(N = 1,964)	12.4% vs. 15.5%	0.78 (0.58–1.05)	0.10

Pneumococcal vaccination (N = 1,950)			0.08
0 doses	19.6%	1.0	
1 dose	17.7%	0.87 (0.52–1.47)	
2 doses	13.9%	0.67 (0.39–1.14)	
3+ doses	13.6%	0.64 (0.44–0.93)	

Influenza vaccination prior to visit (N = 1,950)	12.4% vs. 15.3%	0.78 (0.57–1.06)	0.11

In multivariable models, age and antibiotic use in the past 2 months remained significantly associated with *S. aureus *colonization when adjusted for other individual and community-level demographic characteristics and *S. pneumoniae *colonization (Table 4). Children 6 months-<1 year, 1–<2 years, 2–<3 years, and 3–<4 years of age were significantly less likely to be colonized with *S. aureus *compared to children 5 years and older. Also, children who recently received antibiotics in the past 2 months were less likely to be colonized with *S. aureus*. The addition of other predictors such as breastfeeding, childcare use, diagnosis on day of swab, pneumococcal colonization, number of doses of PCV7 vaccine, and recent influenza vaccination did not change the results of our model. Thus, we present the most parsimonious model in Table 4. Because the number of MRSA isolates was small (N = 13) in our population, we did not examine risk factors for MRSA colonization.

**Table 4:** Multivariate predictors of *Staphylococcus aureus *colonization adjusted for clustering by community (N = 1,707)

	Multivariable OR (95% CI)	P-value
Year of swab		0.71
2003–04	1.0	
2006–07	0.95 (0.71–1.26)	

Age group		<0.001
<6 months	0.61 (0.34–1.08)	
6–<12 months	0.39 (0.24–0.64)	
1–<2 years	0.35 (0.23–0.54)	
2–<3 years	0.45 (0.28–0.73)	
3–<4 years	0.53 (0.33–0.86)	
4–<5 years	0.70 (0.44–1.11)	
≥5 years	1.0	

Female	0.83 (0.63–1.10)	0.20

Race/ethnicity		0.99
White non-Hispanic	1.0	
Black non-Hispanic	0.96 (0.49–1.90)	
Hispanic	0.95 (0.51–1.76)	
Other	0.90 (0.46–1.77)	

Median household income in community		0.89
<$40,000	0.99 (0.51–1.93)	
$40,000–$54,999	1.14 (0.67–1.94)	
$55,000–$69,999	1.11 (0.67–1.84)	
≥$70,000	1.0	

% community members with <high school educational level		0.65
<5%	0.99 (0.53–1.87)	
5–<10%	0.76 (0.45–1.27)	
10–<15%	0.87 (0.57–1.32)	
≥15%	1.0	

Colonization with *Streptococcus pneumoniae*	0.92 (0.67–1.27)	0.62

Antibiotics in past 2 months		0.005
Amoxicillin-clavulanate/cephalosporins/mupirocin/TMP-SMX	0.57 (0.33–0.97)	
Clindamycin/macrolides	0.51 (0.24–1.08)	
Amoxicillin/penicillin	0.54 (0.35–0.84)	
No antibiotics	1.0	

## Discussion

Between 2003–04 and 2006–07, *S. aureus *(15.3% to 14.1%) and MRSA (0.4% to 0.9%) colonization of the nares among children 3 months to <7 years remained stable in the setting of widespread use of the pneumococcal conjugate vaccine in Massachusetts and decreasing colonization with PCV7 strains. [28] Although *S. aureus *colonization was lower among children who were concomitantly colonized with *S. pneumoniae*, this relationship was modest and not statistically significant. Previous studies that found a negative association between these two species documented that the relationship was strongest when limited to *S. pneumoniae *serotypes included in the pneumococcal conjugate vaccine[20,21,29,30] and when limited to MRSA. [29] The lack of statistical significance in our study may be due to the relative rarity of PCV7 serotypes among pneumococci and MRSA among *S. aureus *isolates in our population.

The prevalence of *S. aureus *colonization in young children in Massachusetts is lower than has previously been reported by NHANES (36% among 1–19 year olds), although MRSA colonization in Massachusetts is similar to national estimates (0.9% among 1–19 year olds) from 2001–04. [3] Other cross-sectional studies have found prevalence rates for *S. aureus *and MRSA, ranging from 18.9% to 24.4% and 0.6% to 1.7%, respectively. [31,32] In contrast to our findings, a serial cross-sectional study of nasal carriage among young healthy children conducted in other settings found a rising prevalence of both *S. aureus *(29% to 36%) and MRSA (0.8% to 9.2%) colonization from 2001 to 2004. [33,34] Unlike our study, however, little was known about the potential impact of changing pneumococcal colonization patterns due to PCV7 use on carriage of *S*. *aureus *and MRSA.

Between 2003–04 and 2006–07, *S. aureus *isolates remained susceptible to trimethoprim/sulfamethoxazole, tetracycline, mupirocin, vancomycin and linezolid in 8 communities. However, a smaller proportion of isolates were susceptible to erythromycin and clindamycin in the latter season. Interestingly, antibiotic dispensing for broad-spectrum macrolides, such as clarithromycin and azithromycin, in a similar set of communities in Massachusetts has increased over a similar time period. [35] It is plausible that antibiotic pressure in these communities may have contributed to the emergence of strains such as USA300 with this particular antibiotic resistance phenotype. [36-38]

In multivariate models, *S. aureus *colonization was found to be age-dependent in agreement with other published studies. [20,32,33,39] Recent use of antibiotics was also found to be inversely associated with *S. aureus *colonization similar to prior studies. [39,40] Although we found reduced *S. aureus *colonization following antibiotic use, we may only be seeing a transient decline in *S. aureus *colonization without fully understanding the longer-term impact of antibiotic use and the emergence of resistant organisms, since our study was cross-sectional in design. [41,42] Furthermore, we found that colonization with *S. aureus *was lower in children regardless of the type of antibiotic used. This relationship is intriguing since we postulated that only antibiotics clinically active against *S. aureus *would be inversely associated with colonization as suggested by prior published studies. [39] One possibility is that recent antibiotic use is associated with another characteristic of these individuals that we were not able to measure. It may also be possible that part of the decline is due in part to 11% of the circulating *S. aureus *isolates in our population being susceptible to penicillins. The impact of recent antibiotic use on colonization patterns will be an important area for further study given concerns over the competitive balance between organisms in the nasopharynx. [43]

There are several potential limitations to our study. First, we only included children less than 7 years of age, who may be at lower risk for MRSA colonization than older children. Second, we did not adequately assess all sites of colonization (e.g. skin, throat, rectum), thus underestimating the true prevalence of MRSA colonization, particularly since community-associated MRSA may be more likely than other MRSA or MSSA strains to colonize non-nasal sites. [44] Third, we did not use an enrichment broth to isolate *S. aureus *or MRSA, which may have enhanced detection rates in other studies. Fourth, we only swabbed healthy children in Massachusetts, which is one of the last geographic areas to be hit by the CA-MRSA epidemic. [45] In contrast, significant increases in CA-MRSA disease and colonization have occurred in other parts of the country between 2003–04 and 2006–07. [11,12,31-34,37,46-49] Finally, we did not swab children longitudinally; thus, we were not able to assess whether these risk factors were different for children with transient vs. persistent *S. aureus *carriage.

## Conclusion

We conclude that *S. aureus *and MRSA colonization of the nares remained stable from 2003–04 to 2006–07 among children less than 7 years in Massachusetts communities despite widespread use of pneumococcal conjugate vaccine during these time periods. *S. aureus *nasal colonization varies by age and appears inversely correlated with recent antibiotic use. Continued monitoring of the ecology of colonization patterns among children will be essential to improve our understanding of risk for disease due to *S. aureus *and MRSA in the community.

## Competing interests

SIP has research support from Wyeth, GSK, and Novartis. He had been a member of vaccine advisory boards for Wyeth, GSK, Novartis and Sanofi Pasteur and had received honorarium for such time and efforts. WPH has served as a consultant for Glaxo Smith Kline. All other authors report no conflict of interest.

## Authors' contributions

GML participated in the conception and design of the study, analysis and interpretation of data, and drafting of the manuscript. SSH, SRS, KK, WPH, and ML participated in the analysis and interpretation of data and critical revision of the manuscript. VLH, SIP, AJM, and JAF participated in the acquisition of data, analysis and interpretation of data, and critical revision of the manuscript. All authors read and approved the final manuscript.

## Pre-publication history

The pre-publication history for this paper can be accessed here:

http://www.biomedcentral.com/1471-2334/9/110/prepub
